# Supplementary material for: Does varying the ingestion period of sodium citrate influence blood alkalosis and gastrointestinal symptoms?
Source: PLoS One. 2021 May 17;16(5):e0251808. doi: 10.1371/journal.pone.0251808 (PMC8128256; doi:10.1371/journal.pone.0251808)
Supplement: S1 Table — (DOCX) [file pone.0251808.s001.docx]

**S1 Table.** Pairwise comparisons (mean difference, 95% CI) of post-ingestion session values for blood variables (blood pH, blood [HCO_3_^-^], blood [Na^+^] and blood [Cl^-^]) following ingestion of 500 mg.kg^-1^ BM sodium citrate over 15, 30, 45 or 60 min (*n* = 16 participants, 18 observations per participant per treatment).

|  | **15 min vs 30 min** | **15 min vs 45 min** | **15 min vs 60 min** | **30 min vs 45 min** | **30 min vs 60 min** | **45 min vs 60 min** |
| --- | --- | --- | --- | --- | --- | --- |
| Blood pH | 0.010 (0.005, 0.015) ** | 0.008 (0.002, 0.013) * | 0.009 (0.004, 0.015) ** | -0.002 (-0.008, 0.003) | -0.001 (-0.006, 0.005) | 0.002 (-0.004, 0.007) |
| Blood [HCO_3_^-^] (mmol.L^-1^) | 0.1 (-0.2, 0.5) | 0.0 (-0.3, 0.3) | 0.4 (0.1, 0.8) * | -0.1 (-0.4, 0.2) | 0.3 (0.0, 0.6) | 0.4 (0.1, 0.8) * |
| Blood [Na^+^] (mmol.L^-1^) | 0.2 (-0.1, 0.6) | 0.0 (-0.3, 0.3) | -0.2 (-0.5, 0.2) | -0.2 (-0.6, -0.1) | -0.4 (-0.7, -0.1) * | -0.2 (-0.5, 0.2) |
| Blood [Cl^-^] (mmol.L^-1^) | -0.2 (-0.5, 0.1) | -0.2 (-0.5, 0.1) | -0.4 (-0.7, -0.1) * | 0.0 (-0.3, 0.3) | -0.2 (-0.5, 0.1) | -0.2 (-0.5, 0.1) |

Estimates obtained under a linear mixed model including participants as a random effect and fixed effects: treatment, time (categorical), time by treatment interaction and treatment order. Estimates are presented only for blood variables with non-significant interaction effect. Sidak adjustment for multiple pairwise comparisons. Difference between ingestion periods, * *p* < 0.05, ** *p* < 0.01.
