# Supplementary material for: Does varying the ingestion period of sodium citrate influence blood alkalosis and gastrointestinal symptoms?
Source: PLoS One. 2021 May 17;16(5):e0251808. doi: 10.1371/journal.pone.0251808 (PMC8128256; doi:10.1371/journal.pone.0251808)
Supplement: S2 Table — (DOCX) [file pone.0251808.s002.docx]

**S2 Table.** Pairwise comparisons (mean difference, 95% CI) of curve characteristics for blood pH and blood bicarbonate concentration ([HCO_3_^-^]) following ingestion of 500 mg.kg^-1^ BM sodium citrate over 15, 30, 45 or 60 min (*n* = 16 participants, 18 observations per participant per treatment).

|  |  | | | | | |
| --- | --- | --- | --- | --- | --- | --- |
|  | **15 min vs 30 min** | **15 min vs 45 min** | **15 min vs 60 min** | **30 min vs 45 min** | **30 min vs 60 min** | **45 min vs 60 min** |
| Blood pH | | | | | | |
| Baseline ^^^ | 0.008 (-0.016, 0.032) | 0.022 (-0.002, 0.046) | 0.012 (-0.011, 0.036) | 0.014 (-0.010, 0.038) | 0.004 (-0.020, 0.028) | -0.010 (-0.034, 0.014) |
| iPeak ^†^ | 0.005 (-0.010, 0.019) | -0.004 (-0.019, 0.011) | 0.006 (-0.009, 0.021) | -0.009 (-0.024, 0.006) | 0.001 (-0.013, 0.016) | 0.010 (-0.004, 0.025) |
| iDelta ^†^ | 0.004 (-0.010, 0.019) | -0.004 (-0.019, 0.011) | 0.006 (-0.009, 0.021) | -0.009 (-0.024, 0.006) | 0.001 (-0.013, 0.016) | 0.010 (-0.004, 0.025) |
| Time to iPeak (min) ^†^ | 15 (-54, 84) | -2 (-71, 68) | 73 (-4, 143) * | -17 (-86, 53) | 58 (-11, 128) | 75 (6, 144) * |
| Area under the curve | 6 (-3, 15) | 4 (-4, 13) | 5 (-4, 13) | -2 (-10, 7) | -1 (-10, 8) | 0 (-9, 9) |
| Blood bicarbonate concentration ([HCO_3_^-^]) | | | | | | |
| Baseline (mmol.L^-1^) | -0.1 (-1.5, 1.3) | 0.6 (-0.8, 2.0) | 0.5 (-0.9, 1.9) | 0.6 (-0.8, 2.0) | 0.6 (-0.8, 2.0) | -0.1 (-1.5, 1.3) |
| iPeak (mmol.L^-1^) ^†^ | 0.3 (-0.6, 1.3) | -0.1 (-1.1, 0.9) | 0.4 (-0.6, 1.3) | -0.4 (-1.4, 0.5) | 0.0 (-0.9, 1.0) | 0.5 (-0.5, 1.4) |
| iDelta (mmol.L^-1^) ^†^ | 0.3 (-0.6, 1.3) | -0.1 (-1.1, 0.9) | 0.4 (-0.6, 1.3) | -0.4 (-1.4, 0.5) | 0.0 (-0.9, 1.0) | 0.5 (-0.5, 1.4) |
| Time to iPeak (min) ^†^ | 9 (-57, 75) | 69 (-3, 135) * | 21 (-45, 87) | 60 (-6, 126) | 11 (-55, 77) | -49 (-115, 17) |
| Area under the curve | 165 (-394, 724) | 118 (-442, 677) | 266 (-294, 825) | -47 (-607, 512) | 101 (-459, 660) | 148 (-411, 707) |

^^^ denotes that all mean (95% confidence interval) values were estimated under a linear mixed model (LMM) including treatment as fixed effect and participant as random effect, applied to all outcomes within this table. ^†^ calculated from a smoothed curve for each participant during each individual session. iPeak (the maximum value from each individual session); iDelta (change from baseline to iPeak); Time to iPeak (from completion of ingestion to iPeak). Difference between ingestion periods, * *p* < 0.05.
