# Supplementary material for: Does varying the ingestion period of sodium citrate influence blood alkalosis and gastrointestinal symptoms?
Source: PLoS One. 2021 May 17;16(5):e0251808. doi: 10.1371/journal.pone.0251808 (PMC8128256; doi:10.1371/journal.pone.0251808)
Supplement: S3 Table — (DOCX) [file pone.0251808.s003.docx]

**S3 Table.** Pairwise comparisons (mean difference, 95% CI) of curve characteristics for blood sodium concentration ([Na^+^]), blood chloride concentration ([Cl^-^]) and plasma citrate concentration ([citrate]) following ingestion of 500 mg.kg^-1^ BM sodium citrate over 15, 30, 45 or 60 min (*n* = 16 participants, 18 observations per participant per treatment).

|  |  | | | | | |
| --- | --- | --- | --- | --- | --- | --- |
|  | **15 min vs 30 min** | **15 min vs 45 min** | **15 min vs 60 min** | **30 min vs 45 min** | **30 min vs 60 min** | **45 min vs 60 min** |
| Blood sodium concentration ([Na^+^]) | | | | | | |
| Baseline (mmol.L^-1^) ^^^ | 0.9 (-0.5, 2.4) | 0.7 (-0.7, 2.1) | 0.0 (-1.4, 1.4) | -0.3 (-1.7, 1.2) | -0.9 (-2.4, 0.5) | -0.7 (-2.1, 0.7) |
| iPeak (mmol.L^-1^) ^†^ | 0.0 (-0.9, 0.8) | -0.4 (-1.2, 0.5) | 0.1 (-0.7, 0.9) | -0.3 (-1.1, 0.5) | 0.1 (-0.7, 1.0) | 0.5 (-0.4, 1.3) |
| iDelta (mmol.L^-1^) ^†^ | 0.0 (-0.9, 0.8) | -0.4 (-1.2, 0.5) | 0.1 (-0.7, 0.9) | -0.3 (-1.1, 0.5) | 0.1 (-0.7, 1.0) | 0.5 (-0.4, 1.3) |
| Time to iPeak (min) ^†^ | 17 (-95, 129) | 30 (-82, 142) | 71 (-41, 183) | 13 (-99, 125) | 54 (-58, 166) | 41 (-71, 153) |
| Area under the curve | 115 (-274, 503) | 5 (-384, 393) | -55 (-443, 334) | -110 (-498, 278) | -169 (-557, 219) | -59 (-447, 329) |
| Blood chloride concentration ([Cl^-^]) | | | | | | |
| Baseline (mmol.L^-1^) | 0.1 (-1.0, 1.3) | 0.6 (-0.5, 1.8) | -0.4 (-1.6, 0.7) | 0.5 (-0.7, 1.7) | -0.6 (-1.7, 0.6) | -1.1 (-2.2, 0.1) |
| iPeak (mmol.L^-1^) ^†^ | -0.9 (-2.0, 0.3) | -0.8 (-2.0, 0.3) | -0.5 (-1.6, 0.7) | 0.0 (-1.1, 1.2) | 0.4 (-0.8, 1.6) | 0.4 (-0.8, 1.6) |
| iDelta (mmol.L^-1^) ^†^ | -0.9 (-2.0, 0.3) | -0.8 (-2.0, 0.3) | -0.5 (-1.6, 0.7) | 0.0 (-1.1, 1.2) | 0.4 (-0.8, 1.6) | 0.4 (-0.8, 1.6) |
| Time to iPeak (min) ^†^ | 98 (-18, 177) * | 86 (-6, 166) * | 105 (25, 185) * | -11 (-91, 69) | 8 (-72, 87) | 19 (-61, 99) |
| Area under the curve | -68 (-576, 440) | -87 (-595, 421) | -164 (-672, 344) | -19 (-527, 489) | -96 (-604, 412) | -77 (-585, 431) |
| Plasma citrate concentration ([citrate]) | | | | | | |
| Baseline (µmol.L^-1^) | 10.5 (-17.7, 38.7) | 11.3 (-15.7, 38.3) | 12.6 (-14.4, 39.6) | 0.8 (-27.4, 29.1) | 2.1 (-26.1, 30.3) | 1.3 (-25.7, 28.3) |
| iPeak (µmol.L^-1^) ^†^ | 7.5 (-41.5, 56.5) | 17.9 (-29.0, 64.8) | -6.4 (-53.4, 40.6) | 10.4 (-38.2, 59.0) | -13.9 (-62.5, 34.7) | -24.3 (-70.7, 22.1) |
| iDelta (µmol.L^-1^) ^†^ | 7.5 (-41.5, 56.5) | 17.9 (-29.0, 64.8) | -6.4 (-53.4, 40.6) | 10.4 (-38.2, 59.0) | -13.9 (-62.5, 34.7) | -24.3 (-70.7, 22.1) |
| Time to iPeak (min) ^†^ | 17 (-11, 45) | 17 (-11, 45) | 23 (-5, 50) | 0 (-28, 28) | 6 (-22, 33) | 6 (-22, 33) |
| Area under the curve | 7219 (-2323, 16762) | 14910 (-5368, 24453) ** | 7021 (-2742, 16785) | 7691 (-1852, 17233) | -198 (-9962, 9565) | -7889 (-17653, 1875) |

^^^ denotes that all mean (95% confidence interval) values were estimated under a linear mixed model (LMM) including treatment as fixed effect and participant as random effect, applied to all outcomes within this table. ^†^ calculated from a smoothed curve for each participant during each individual session. iPeak (the maximum value from each individual session); iDelta (change from baseline to iPeak); Time to iPeak (from completion of ingestion to iPeak). Difference between ingestion periods, * *p* < 0.05, ** *p* < 0.01.
