# Supplementary material for: Does varying the ingestion period of sodium citrate influence blood alkalosis and gastrointestinal symptoms?
Source: PLoS One. 2021 May 17;16(5):e0251808. doi: 10.1371/journal.pone.0251808 (PMC8128256; doi:10.1371/journal.pone.0251808)
Supplement: S4 Table — (DOCX) [file pone.0251808.s004.docx]

**S4 Table.** Pairwise comparisons (mean difference, 95% CI) of palatability (*n* = 16 participants) and urinary excretion values (*n* = 8 participants) following ingestion of 500 mg.kg^-1^ BM sodium citrate over 15, 30, 45 or 60 min.

|  |  | | | | | |
| --- | --- | --- | --- | --- | --- | --- |
|  | **15 min vs 30 min** | **15 min vs 45 min** | **15 min vs 60 min** | **30 min vs 45 min** | **30 min vs 60 min** | **45 min vs 60 min** |
| Palatability | | | | | | |
| Palatability score (mean, 95% confidence intervals) | -0.6 (-1.4, 0.2) | -0.1 (-0.9, 0.7) | -0.3 (-1.0, 0.5) | 0.5 (-0.3, 1.3) | 0.4 (-0.4, 1.2) | -0.1 (-0.9, 0.7) |
|  |  |  |  |  |  |  |
| Urinary excretion | | | | | | |
| Urinary [citrate] (µmol.L^-1^) | 137.2 (-186.0, 460.4) | 176.6 (-143.9, 497.1) | 54.8 (-268.4, 378.0) | 39.4 (-283.8, 362.6) | -82.4 (-402.9, 238.1) | -121.8 (-445.0, 201.4) |
| Total excreted citrate (mg) | -63.9 (-204.1, 76.3) | 39.0 (-100.0, 178.1) | -27.6 (-167.8, 112.6) | 102.9 (-37.3, 243.1) | 36.3 (-102.8, 175.4) | -66.6 (-206.8, 73.6) |
| Delta excreted citrate (total excreted citrate minus pre-testing citrate (mg)) | -63.9 (-204.1, 76.3) | 39.0 (-100.0, 178.1) | -27.6 (-167.8, 112.6) | 102.9 (-37.3, 243.1) | 36.3 (-102.8, 175.4) | -66.6 (-206.8, 73.6) |
|  |  |  |  |  |  |  |
| Total urine excreted (mL) | -188.1 (-488.1, 111.8) | -140.7 (-438.2, 156.8) | -133.5 (-433.5, 166.4) | 47.5 (-25.5, 347.4) | 54.6 (-242.9, 352.1) | 7.1 (-292.8, 307.1) |
